# The Effect of Violent Virtual Avatar Experience on Players' Response Inhibition to Angry Expressions and Its Cognitive Neural Mechanisms

**DOI:** 10.1027/1618-3169/a000662

**Published:** 2026-04-23

**Authors:** Lijun Sun, Xuelian Li, Shiyi Li, Xue Zhang, Yajing Si, Hongxing Zhang

**Affiliations:** ^1^ School of Psychology, Henan Medical University, Xinxiang, PR China

**Keywords:** virtual avatar, response inhibition, angry expressions, N2, P3

## Abstract

**Abstract:** Response inhibition may be a flexible resource that is usually in a dormant state but can be awakened in particular contexts, such as angry expressions. Few existing studies have explored the influence of violent video games on players' response inhibition to angry expressions from the perspective of virtual avatars. The aim of this study was to explore the effect of violent virtual avatar experience on players' response inhibition to angry expressions and its cognitive neural mechanisms. Eighty-four players (42 participants each with high and low violent virtual avatar experience) were selected to complete the emotional Go/No-go task, during which participants' EEG were recorded. Results indicated that facing angry expressions players with high violent virtual avatar experience showed a greater No-go P3 effect than those with low violent virtual avatar experience. These results suggest that facing angry expressions players with high violent virtual avatar experience show superiority in response inhibition, and this superiority exists in the later stage of response inhibition, which is closely related to the actual inhibition of the motor system.


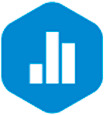


Since the first video games emerged in the 1970s, the popularity of video games has continuously risen (Gough, 2019). Among underage internet users, about 60% frequently engage in online video games, and 13.2–42% spend more than 2 h a day playing games (China Internet Network Information Center, 2023; Entertainment Software Association, 2015). According to statistics, among the most popular online video games, 89% of video games contain some content of violence (Siyez & Baran, 2017). Violent video games can have negative impacts on players' cognition, emotions, and behaviors. Among them, the influence of violent video games on players' cognition has always been a hot topic in psychological research (Ritchie et al., 2024; Zhang et al., 2021).

Response inhibition is an important cognitive component, which refers to the ability to suppress inappropriate and unwanted actions (Luijten et al., 2011). Previous studies have investigated the impact of violent video games on response inhibition (Colzato et al., 2013; Miedzobrodzka, et al., 2021), but few studies have focused on the influence of avatars, which are core elements of violent video games, on players' response inhibition. Virtual avatar is virtual self-presentation mode that players can see and manipulate in real time in video games (Peña et al., 2009). Gamma data suggest that avatar-based games have become mainstream in the online gaming space in recent years since the epidemic (Gamma Data, 2022), and that players have become more addicted to the genre (Bekir & Çelik, 2020). Existing behavioral studies using stop-signal task and Go/No-go task showed that long-term avatar exposure in violent video game did not lead to any significant negative effects on players' response inhibition (Kühn et al., 2019; Metcalf & Pammer, 2014). Response inhibition may be a flexible resource that is usually in a dormant state but can be awakened in particular contexts, such as hostile situations, because facing hostile situations, individuals will have more impulsiveness, and at this time, the participation of response inhibition is needed (Wilkowski & Robinson, 2010). Angry expressions are often used as stimuli to represent hostile situations (Lievaart et al., 2016). Future research needs to explore the effect of violent avatar experience on players' response inhibition to angry expressions and reveal its cognitive neural mechanism.

Event-related potential (ERP) technique offers high temporal resolution, which makes it sensitive to rapid changes in brain activity that occur within milliseconds after stimulus onset (Otten & Rugg, 2004). The technique has been frequently used to explore the cognitive neural mechanisms of response inhibition (Lee & Tong, 2024; Wang et al., 2023). In ERP studies, the emotional Go/No-go paradigm has been frequently used to measure individual response inhibition (Magee et al., 2023; Sun et al., 2023). In this task, the emotional stimulus is used as either a Go or No-go stimulus, and participants are asked to respond to the Go stimulus and not to the No-go stimulus. Two major ERP components, the N2 and P3, are elicited in this tasks (Magee et al., 2023; Zhang et al., 2023). N2, a frontally distributed negative waveform at 200–400 ms after stimulus is presented, is significantly greater for No-go relative to Go condition, the so-called No-go N2 effect. This effect reflects the early stage of conflict monitoring before a correct response (Donkers & Van Boxtel, 2004). P3, a frontocentrally distributed positive waveform at 300–700 ms after stimulus onset, is also significantly greater for No-go relative to Go condition, the so-called No-go P3 effect. This effect reflects the later stage of actual inhibitory control that is closely related to the actual inhibition of the motor system in the premotor cortex (Smith et al., 2008). This effect also reflects the attentional resources allocated to infrequent, salient, and inhibitory stimuli (e.g., No-go stimuli; Littel et al., 2012).

Stockdale et al. (2017) measured response inhibition using an affective stop-signal task, which is different from the Go/No-go paradigm (Verbruggen & Logan 2008). Stockdale et al. (2017) measured response inhibition using the N2 and P3 in the Stop trials and believed that in the stop-signal task, the reduced N2/P3 amplitude after the onset of the stop-signal indicates that less neural resources were recruited to inhibit behavior, representing superior inhibitory control. Another study suggested that larger Stop-N2 and Stop-P3 amplitudes are related to better cognitive control (Camfield et al., 2018; Folstein & Van Petten, 2008; Yu et al., 2022). Wüllhorst et al. (2025) used the successful inhibition-P3 effect (successful stop vs. go trials) as an indicator of response inhibition, and the larger this effect, the stronger the response inhibition. Thus, it can be seen that there are inconsistent research results regarding how the size of Stop-N2 and Stop-P3 amplitudes reflect the level of response inhibition in the stop-signal task. Unlike the stop-signal task, this study adopted the Go/No-go paradigm. In the Go/No-go paradigm, the N2 and P3 difference waves (the difference in amplitude between the No-go and Go waves) are commonly used to measure the degree of response inhibition (Sun et al., 2020; Sun et al., 2023). Existing ERP studies using Go/No-go paradigm revealed significant reductions in No-go N2 effect in the video game addiction (VGA) group, which indicate that VGA subjects have lower early stages of response inhibition (Fathi et al., 2024). Some studies showed that individuals with Internet addiction disorder (IAD) showed reduced No-go N2 effect (Dong et al. 2010; Zhou et al. 2010) and increased No-go P3 effect (Dong et al. 2010), which suggest that the IAD individuals had lower activation in the conflict detection stage than the normal ones; thus, they had to engage in more cognitive endeavors to complete the inhibition task in the late stage. Another study found No-go N2 effect and No-go P3 effect did not differ between excessive gamers and controls (Littel et al., 2012). Existing ERP studies using stop signal paradigm found that under implicit conditions, facing fearful expressions, frequent players of violent video games had superior inhibitory control (Stockdale et al., 2017). In the implicit task, the emotion salience induced by facial expressions is less significant and has less effect on response inhibition. In explicit tasks, facial expressions can induce more emotional salience and have a greater impact on response inhibition. At the same time, fear is an indirect threat and anger is a direct threat (Hortensius et al., 2016). Therefore, further research is needed to explore the impact of violent virtual avatar experiences on players' response inhibition to angry expressions and its underlying cognitive neural mechanisms from explicit perspective.

## The Current Study

The General Aggression Model (GAM) holds that the occurrence of aggressive behavior involves both distal factors and proximate factors (Allen et al., 2018). Exposure to violent virtual avatar is a distal factor. Repeated use of virtual avatars to engage in violent behavior can affect individuals' personality, causing them to have aggression-related knowledge structures built in memory. Over time, desensitization to other violent stimuli occurs (Carnagey et al., 2007; Staude-Müller et al., 2008), which influences subsequent episodes of aggression at the proximate level by altering person and situation factors. This thereby affects the players' cognition, and further increases the likelihood of aggression. This study aimed to explore the effects of violent virtual avatar experience on players' response inhibition to angry expressions and its cognitive neural mechanism from explicit perspective, which helps us to have a more comprehensive understanding of the impact of violent avatar use on players' cognition, deepen our understanding of the relationship between violent media and players' brain, and thereby understand the occurrence of aggressive behaviors. In line with the GAM and violent media desensitization model (Carnagey et al., 2007), studies showed players with high violent video game experience showed worse recognition and desensitization to angry expressions (Miedzobrodzka, et al., 2021; Miedzobrodzka et al., 2022). According to dual competition model (DCM, Pessoa, 2009), which holds that cognitive resources are finite, their desensitization to angry expressions could cause them to pay less attention to angry expressions, which might lead to better response inhibition through increasing the availability of attentional resources. Therefore, this study examined whether players with high violent virtual avatar experience would show better response inhibition when facing angry facial expressions, as indicated by fewer commission errors in the No-go task, a larger No-go N2 effect, and a greater No-go P3 effect.

## Method

### Participants

Based on the existing literature (Sun et al., 2025), G * Power 3.1 software was used (with effect size to 0.25, power set to 80%, and alpha level set to .05). The sample size was calculated in each group to be 12. Convenience sampling was used to select 400 freshmen (200 males) as the participant. Referring to the existing literature (Sun et al., 2020), players with high violent virtual avatar experience met the following criteria: The score of the Violent Virtual Avatar Experience Questionnaire ranked in the top 27%, and had a gaming experience of at least 3 years, with a gaming duration of more than one hour per session and playing games for at least 10 h per week. Players with low violent virtual avatar experience met the following criteria: The score of the Violent Virtual Avatar Experience Questionnaire was in the bottom 27%, and did not play or play video games less frequently. Finally, 108 players with high violent virtual avatar experience (55 males) and 108 players with low violent virtual avatar experience (56 males) were selected. Based on the principles of gender balance and voluntariness, ultimately 43 players with high violent virtual avatar experience (26 males) and 43 players with low violent virtual avatar experience (22 males) were randomly selected, aged between 17 and 20 years old (M = 18.89, *SD* = 1.11). There was no significant difference in age (*t* (82) = 0.485, *p* = .629) between players with high violent virtual avatar experience (*M* = 18.83, *SD* = 1.12) and players with low violent virtual avatar experience (*M* = 18.95, *SD* = 1.12). The violent virtual avatar experience of players with high violent virtual avatar experience (*M* = 20.86, *SD* = 5.22) was significantly higher than that of players with low violent virtual avatar experience (*M* = 2.47, *SD* = 2.47; *t* (82) = 2.27, *p* = .026). Two participants (one in players with high violent virtual avatar experience) were excluded due to more than 50% of the trials containing ERP artifacts.

### Materials and Procedure

#### Violent Virtual Avatar Experience Questionnaire

Referring to Violent Game Experience Questionnaire compiled by Gentile et al. (2004), we have developed our own Violent Virtual Avatar Experience Questionnaire. The questionnaire requires participants to write down three favorite game avatars and evaluate them, including the frequency of playing game avatars (1 = once a month or less ∼5 = five times a week or more), and the level of violence of game avatars (1 = no bloody or violent ∼6 = very bloody or violent). There are a total of six items. Experience level of violent virtual avatars = ∑(violence level of game avatars multiplied by game frequency)/3. The questionnaire score ranges from 1 to 30 points, and the higher the score, the higher the participant's experience with violent virtual avatars. The Cronbach's α in this study was 0.91.

#### Emotional Face Stimuli

The experimental materials consisted of 72 images of angry faces (36 females) and 72 images of neutral faces (36 females) randomly selected from the Chinese Facial Affective Picture System, which is a standardized facial expression system established for the group of college students (Wang & Luo, 2005).

#### Emotional Go/No-go Task

**Figure 1:**
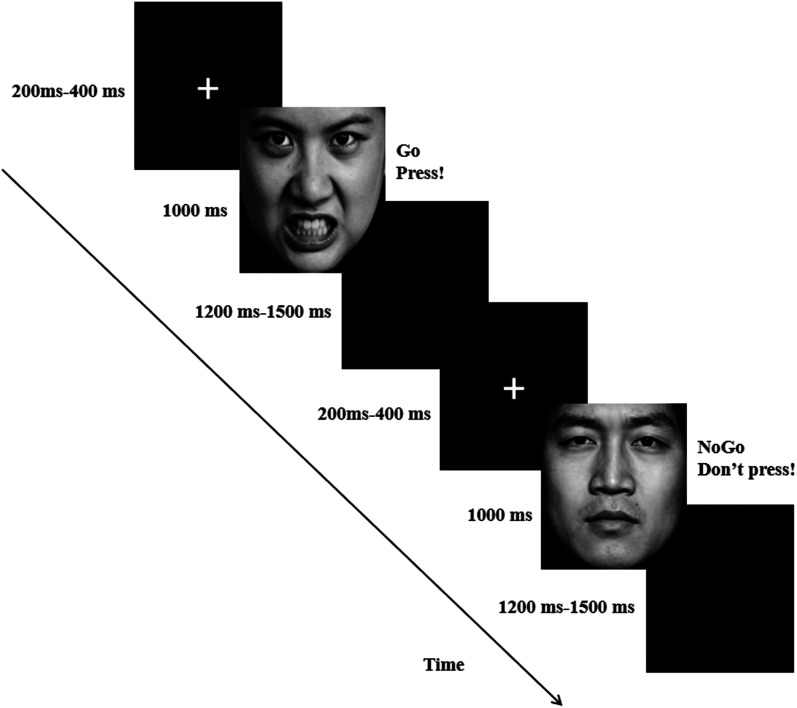
Flow map of emotional Go/No-go task in which angry expressions served as Go cues and neutral expressions were No-go cues. Angry and neutral faces were selected from the Chinese Facial Affective Picture System (Wang & Luo, 2005).

An emotional Go/No-go task was used to measure response inhibition to angry expressions. Angry and neutral pictures were used as frequent Go or infrequent No-go stimuli. In each trial, a fixation point was presented for 200–400 ms (random floats), followed by a face picture for 1,000 ms, and then a black screen for 1,200–1,500 ms (random floats), as shown in Figure 1. Participants were asked to respond quickly and accurately to Go stimuli, not to respond to No-go stimuli. In the stimulus interface, if the participant responded, the blank screen interface was entered, and if the participant did not respond within 1,000 ms, the stimulus interface was presented for 1000ms and then the blank screen interface was presented. The task was divided into two blocks: angry Go/neutral No-go, neutral Go/angry No-go. Each block completed 168 Go trials (72 pictures were repeated) and 72 No-go trials, a total of 240 trials, of which 30% were No-go and 70% were Go trials. In each block, Go trials always preceded No-go trials to induce prepotent conflict and motor responses during response inhibition. The formal experiment consisted of 480 trials, with a rest for every 160 trials.

#### Electrophysiological Recording and Analysis

The electroencephalographic (EEG) data were recorded using the 64-channel Neuroscan system (Neuroscan SynAmps2; Neuroscan Inc., Sterling, VA, USA) according to the extended international 10/10 system. Electrode impedance was kept below 5 kΩ. All signals were digitized with a sampling rate of 500 Hz and 32 bit A/D conversion with a band-pass filter of 0.05–100 Hz.

The EEG data were offline-referenced to the average of the left and right mastoids. A filter with a bandpass of 0.15–30 Hz was adopted to remove high-frequency noise. Independent Component Analysis (ICA) was used to reject the eye movement artifacts (blinks and eye movements). Epochs with a voltage exceeding ±75 µV were excluded. Stimulus-locked epochs were computed. Each epoch was segmented from 200 ms prior to stimulus onset to 1,000 ms after stimulus onset, and those from 200 ms before stimulus onset served as a baseline.

For the N2 and P3 components, the base-peak ERP amplitudes were calculated. Segments with incorrect responses (misses for Go trials or false alarms for No-go trials) were eliminated from the analyses. The N2 and P3 were defined as the peak amplitude within the 300–500 ms and 480–640 ms range after the stimulus was present. Referring to the existing literature (Euser & Franken, 2012; Sun et al., 2020) and the ERP grand average waveforms, we selected F3, Fz, F4, FC3, FCz, FC4, and F3; Fz, F4, FC3, FCz, FC4, C3, Cz, and C4, respectively, for statistical analysis of the N2 and P3 amplitudes.

### Statistical Analyses

#### Behavioral Analyses

To investigate the effect of violent virtual avatar experience on players' response inhibition to angry expressions, taking gender as the covariate, a 2 (Group: players with high violent virtual avatar experience, players with low violent virtual avatar experience) ×2 (Emotion: anger, neutral) ×2 (Trial Type: Go, No-go) repeated-measures ANOVA was conducted to analyze the error rate (including percentage of commission errors and percentage of omission errors). Taking gender as the covariate, a 2 (Group: players with high violent virtual avatar experience, players with low violent virtual avatar experience) ×2 (Emotion: Anger, Neutral) repeated-measures ANOVA was conducted to analyze the reaction time of correct Go trial. Data with reaction times outside 150–1500 ms were excluded.

#### ERP Analyses

To evaluate the effect of violent virtual avatar experience on players' response inhibition to angry expressions, taking gender as the covariate, a Group (players with high violent virtual avatar experience, players with low violent virtual avatar experience) × Emotion (anger, neutral) × Electrode (N2: F3, Fz, F4, FC3, FCz, and FC4; P3: F3, Fz, F4, FC3, FCz, FC4, C3, Cz, and C4) repeated-measures ANOVA was performed for the stimulus-locked N2 and P3 difference waves, which were defined the amplitude on No-go conditions minus the amplitude on Go conditions. Greenhouse-Geisser corrections were adopted where appropriate. All significant ANOVA effects were further analyzed using Bonferroni corrected post hoc *t*-tests. Simple effects analysis was utilized only for interactions including the between-subject factor of group.

## Results

### Behavioral Results

**Figure 2:**
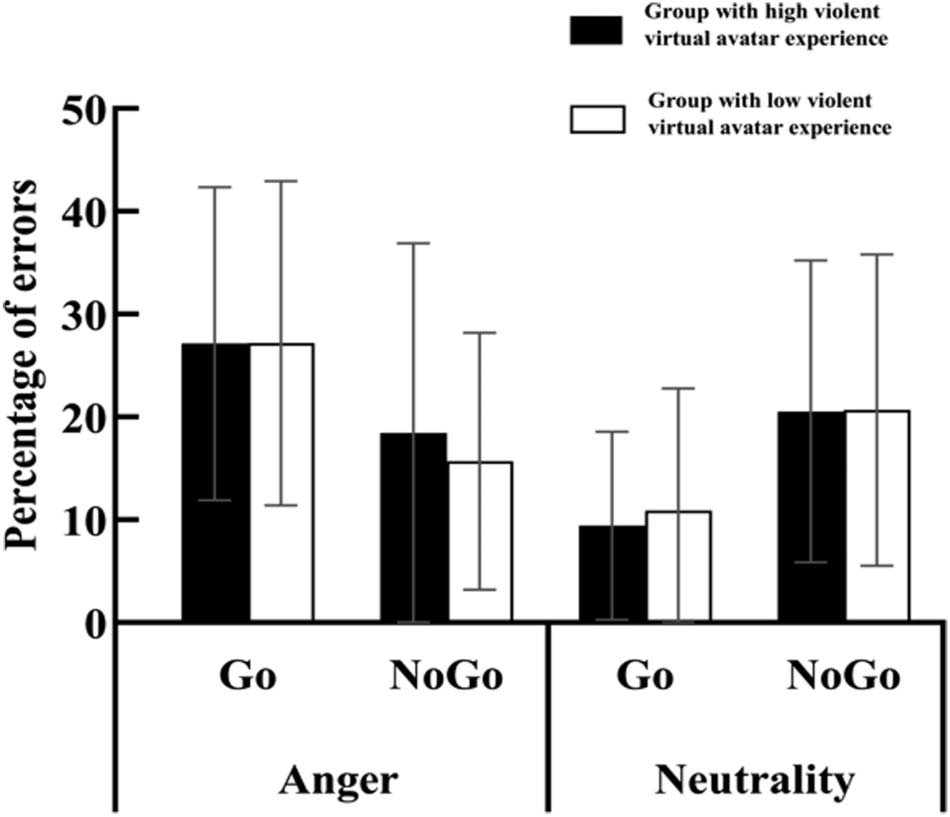
Error rates during the emotional Go/No-go task by group.

**Figure 3:**
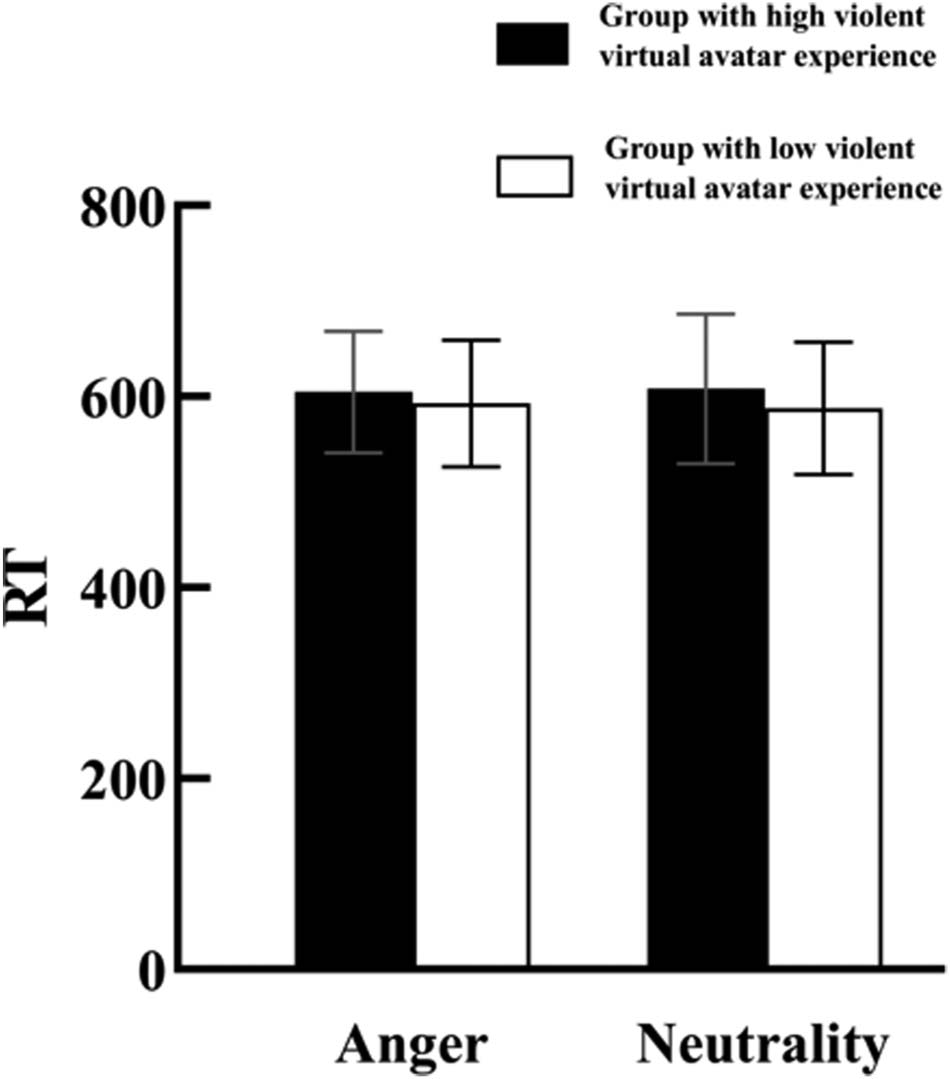
Reaction time of correct Go trials during the emotional Go/No-go task by group.

The error rate and reaction time of group with high and low violent virtual avatar experience are shown in Table 1, Figures 2, and 3. The error rate results showed that the main effect of trial type was significant (F (1,81) = 14.25, *p* < .001, η2 = 0.150). The error rate for Go trials (22.11 ± 1.30%) was significantly higher than that for No-go trials (15.39 ± 1.09%). The interaction between emotions and trial type was significant (F (1,81) = 35.12, *p* < .001, η2 = 0.302). In the Go trials, the error rate for angry expressions was higher than that for neutral expressions (27.15 ± 1.65% vs. 17.06 ± 1.73%, *p* < .001), while in the No-go trials, there was no significant difference in error rates between the angry expressions and neutral expressions (10.17 ± 1.15% vs. 20.60 ± 1.62%, respectively, *p* = .137).

**Table 1:** Behavioral results of emotional Go/No-go tasks in group with high violent virtual avatar experience and group with low violent virtual avatar experience.

Groups	Variables	Emotion			
		Anger		Neutrality	
		*M*	*SD*	*M*	*SD*
Group with high violent virtual avatar experience	CE (%)	9.52	1.63	20.37	2.29
	OE (%)	26.85	2.33	18.48	2.45
	Go RT (ms)	604.18	10.03	608.96	11.33
Group with low violent virtual avatar experience	CE (%)	10.82	1.63	20.83	2.29
	OE (%)	27.44	2.33	15.65	2.45
	Go RT (ms)	593.23	10.03	586.61	11.33
*Note*. CE = percentage of commission errors; OE = percentage of omission errors; ACC = accuracy rate; RT = reaction time for correct Go trials; ms = milliseconds.					

We observed a nonsignificant main effect of group (F(1,81) = 0.004, *p* = .947, η^2^ = 0.001), emotion (F(1,81) = 0.006, *p* = .938, η^2^ = 0.001) and sex (F(1,81) = 0.40, *p* = .241, η^2^ = 0.017). We observed a nonsignificant interaction effect of group and emotion (F(1,81) = 1.26, *p* = .265, η^2^ = 0.015), and group and trial type (F(1,81) = 0.42, *p* = .52, η^2^ = 0.005). We also observed a nonsignificant interaction effect of group, emotion, and trial type (F(1,81) = 0.14, *p* = .71, η^2^ = 0.002).

The reaction time results showed that the main effects of emotion was significant (F(1,81) = 4.10, p = .046, η^2^ = 0.048). The reaction time for angry expressions (598.71 ± 7.08 ms) was significantly higher than that for neutral expressions (597.78 ± 8.00 ms). The main effects of group (F(1,81) = 1.54, *p* = .218, η2 = 0.019), and interaction between group and emotion (F(1,81) = 0.66, *p* = .42, η^2^ = 0.008) were not significant.

### Go/No-go Difference ERP Waveforms

#### N2 Difference Waves

The main effect of emotion was significant (F (1,81) = 5.89, *p* = .017, η^2^ = 0.068), indicating difference amplitude induced by angry expressions (0.875 ± 0.420 μV) was significantly smaller than that induced by neutral faces (−1.420 ± 0.499 μV). There were no significant main effects of electrode (F (5,405) = 1.17, *p* = .325, η^2^ = 0.014) and sex (F (1,81) = 0.24, *p* = .625, η^2^ = 0.003).

Regarding the group effect, the main effect of group was nonsignificant (F(1,81) = 0.05, *p* = 817, η^2^ = 0.001). The interaction effect of group and emotion (F(1, 81) = 0.002, p = .966, η^2^ = 0.001); group and electrode (F(5, 405) = 0.49, *p* = .787, η^2^ = 0.006); group, emotion, and electrode (F(5, 405) = 1.32, *p* = .255, η^2^ = 0.016) was nonsignificant.

#### P3 Difference Waves

The main effect of electrode was significant (F(8,648) = 4.10, *p* < .001, η^2^ = 0.048), indicating difference amplitude at FCz was significantly greater than that at other sites (*p*s < 0.001), and difference amplitude at Cz was significantly greater than that at F3, F4, FC4, C4 sites (*p*s < 0.001). The main effect of emotion was significant (F(1,81) = 7.05, *p* = .010, η^2^ = 0.080), indicating difference amplitude induced by angry expressions (3.609 ± 0.439 μV) was significantly greater than that induced by neutral expressions (0.786 ± 0.547 μV). The main effect of sex was nonsignificant (F (1,81) = 0.01, *p* = .929, η^2^ = 0.001).

**Figure 4:**
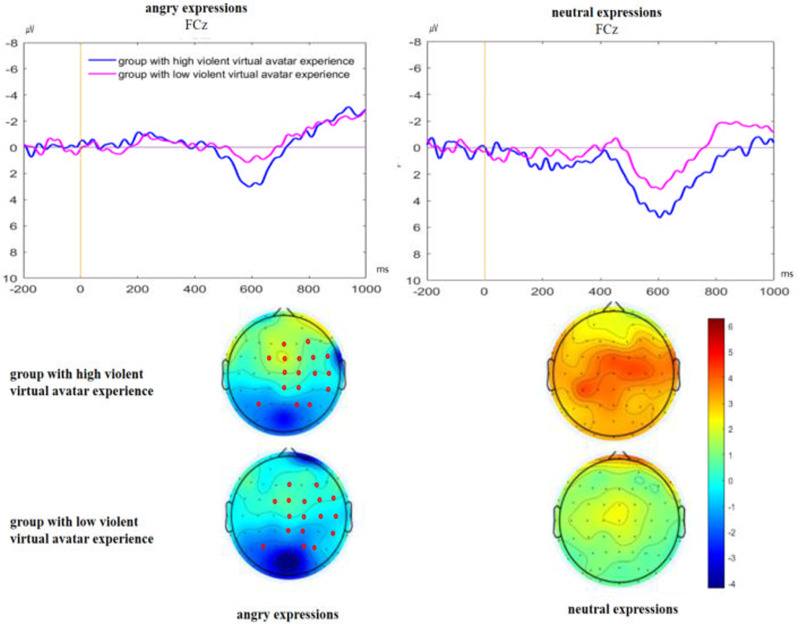
P3 difference waves of No-go subtracted from Go trials at the FCz site and corresponding scalp topography of the two groups in angry and neutral condition. The red marks indicate the electrode points where there are intergroup differences facing angry of neutral expressions.

Regarding the group effect, the main effect of group was significant (F(1,81) = 4.96, *p* = .029, η^2^ = 0.058), indicating the amplitude of players with high violent virtual avatar experience (2.978 ± 0.495 μV) was significantly higher than that of players with low violent virtual avatar experience (1.417 ± 0.495 μV), as shown in Figure 4. The interaction effect of group and emotion (F(1, 81) = 0.34, *p* = .560, η^2^ = 0.004); group and electrode (F(8, 648 ) = 0.70, *p* = .693, η^2^ = 0.009); group, emotion, and electrode (F(8, 648) = 1.39, *p* = .200, η^2^ = 0.017) was nonsignificant.

## Discussion

The present study aimed to investigate the effects of violent virtual avatar experience on players' response inhibition to angry expressions and its cognitive neural mechanism. Results showed that, as a whole, players showed larger P3 amplitudes for No-go trials compared to Go trials, indicating that the affective Go/No-go task was valid in allowing participants to create a dominant response which is difficult to suppress (Euser & Franken, 2012).

Existing behavioral studies using nonemotional stop-signal task and Go/No-go task showed that long-term avatar exposure in violent video game did not lead to any significant negative effects on players' response inhibition (Metcalf & Pammer, 2014). Based on previous studies, this study used emotional Go/No-go task to explore the effect of violent virtual avatar experience on players' response inhibition to angry expressions. The behavioral analyses indicated that when facing angry expressions, players with high violent virtual avatar experience showed the same commission errors on the No-go trials as players with low violent virtual avatar experience. The results of this study are inconsistent with the results of previous behavioral studies which took frequent players of violent video game as the research object using emotional Stop Signal Task (Miedzobrodzka et al., 2021). This study did not showed any between-group difference. First, changes in brain activity may not necessarily be matched by a corresponding behavioral response. Behavioral responses may be influenced by certain moderating factors, such as social desirability bias or the inhibitory effect of laboratory settings on response inhibition, leading to the idea that different cognitive, emotional, and motivational processes produce similar behavioral outputs (Wiswede et al., 2011). Second, ERP technology is more sensitive than behavioral experiments and this high sensitivity gives ERP a high degree of accuracy in detecting brain activity (Euser & Franken, 2012). Further studies could use ERP technique to explore the effect of violent virtual avatar experience on players' response inhibition to angry expressions from the electrophysiological level.

Studies found excessive internet user showed reduced No-go N2 effect compared with controls in a nonemotional Go/No-go paradigm, indicating reduced response inhibition in this population (Dong et al., 2010; Yuan et al., 2008). This study took players with high and low violent virtual avatar experience in the normal population as the research object using emotional Go/No-go paradigm. N2 difference wave results showed that players with high violent virtual avatar experience showed the same No-go N2 effect as the players with low violent virtual avatar experience, which indicated that there was no abnormality in conflict monitoring for players with high violent virtual avatar experience. The present results are a useful supplement to the existing research.

This study explored the cognitive neural mechanism of response inhibition to angry expressions in players with high violent virtual avatar experience in explicit tasks. Difference waves analysis showed the main effect of group is significant, indicating the difference amplitude of players with high violent virtual avatar experience was significantly higher than that of players with low violent virtual avatar experience. These results suggested facing angry expressions, players with high violent virtual avatar experience showed a larger No-go P3 effect than players with low violent virtual avatar experience. Differential wave topographic map results also indicated that facing angry expressions, there are significant intergroup differences in brain region activation. The results of this study are consistent with ERP studies on the effect of exposure to violence on the response inhibition to negative emotions in individuals under implicit conditions (Stockdale et al., 2015; Stockdale et al., 2017). This suggests that players with high violent virtual avatar experience have superiority in response inhibition to angry expressions, and this superiority exists in the later stage of response inhibition, which is closely related to the actual inhibition of the motor system, rather than the early stages of conflict monitoring. According to violent media desensitization model (Carnagey et al., 2007), players with high violent virtual avatar experience are chronically exposed to hostile situations, and will be desensitized to hostile situations (Stockdale et al., 2015; Yang et al., 2024; Zhang et al., 2016). This desensitization to angry expressions could cause them to pay less attention to angry expressions, which might lead to better response inhibition to angry expressions. In addition, angry expression is a strong signal of threat or conflict. For players who embody violence in the game, they may often be in a “high conflict simulation situation” during the game process. In such an environment, maintaining a high level of vigilance and restraint toward threat signals might be a learned, more adaptive survival strategy. For example, in fighting games, when seeing the opponent “getting angry” (entering a rage state), players need to remain extremely calm and judge the timing to block or counterattack instead of blindly charging forward. Therefore, the exposure to violent virtual avatars may refine the cognitive process of players, teaching them to call upon stronger cognitive control resources to suppress instinctive impulses when facing specific high-risk signals (such as anger), to achieve better game results (such as victory; Stockdale et al., 2017). In addition, No-go P3 effect also reflects the amount of attentional allocation or neural resources devoted to inhibition. When no difference on behavioral level, but larger P3 effect was found, it might be because players with high violent virtual avatar experience is using more effort to inhibit their reactions.

According to GAM, exposure to violent virtual avatar can affect individuals' cognition, thereby increase the occurrence of aggressive behavior (Allen et al., 2018). This study found that players with high violent virtual avatar experience showed enhanced response inhibition to angry expressions. Existing research discovered that players with high violent virtual avatar experience displayed increased aggressive behavior, mainly manifested in the decision phase and the outcome feedback phase of players' aggressive behavior (Sun et al., 2025). Therefore, we can infer that the influence of violent media is not a simple, linear “making people violent,” but rather reshapes the individuals' processing mode and response strategy for specific social/emotional cues. Response inhibition is an “instrumental” executive function; it does not determine the purpose of the behavior itself, but only determines the precision and control of the behavior. Exposure to violent virtual avatar shapes a “strategic aggressor” mode. On the one hand, it activates aggressive cognition, making individuals more inclined to choose aggression as a means of problem-solving (decision-making stage); on the other hand, through high-conflict training in the simulated environment, it strengthens the ability to implement precise and controlled behaviors when facing threat signals (such as anger; enhanced response inhibition). This controlled aggression is often more successful, and the positive feedback of success further consolidates the tendency of the aggression decision, ultimately leading to more frequent, determined, and effective aggressive behavior in real life when individuals are in competitive or provoked situations. Therefore, “response inhibition” and “aggressiveness” cannot be simply regarded as mutually exclusive opposites. In specific situation, high control can be combined with high aggression tendency to produce a more adaptable and destructive aggressive behavior pattern. The results of this study further improve GAM.

Furthermore, this study found that the main effect of emotion was significant, indicating N2 difference amplitude induced by angry expressions was significantly smaller than that induced by neutral faces. Anger represents hostility (Wilkowski, 2012), and it is a direct threat. According to the Integrative Cognitive Model (ICM) of Reactive Aggression (Wilkowski & Robinson, 2010), facing angry expressions, individuals will automatically make hostile interpretations becoming even angrier, thereby reducing response inhibition and leading to aggressive behavior. Therefore, the response inhibition in the anger condition is lower than that in the neutral condition. This study also found that the main effect of emotion was significant, indicating P3 difference amplitude induced by angry expressions was significantly greater than that induced by neutral expressions. The motivational direction of anger is approaching (Carver & Harmon, 2009; Du, 2012). Anger Go is consistent with individuals' motivational direction, and participants require fewer cognitive resources. Anger No-go is inconsistent with the individuals' motivational direction, and participants require more cognitive resources. Therefore, the difference between No-go and Go in the anger condition is greater than that in the neutral condition. The results of this study indicate that the emotional effects of response inhibition mainly manifest in the early stage of response inhibition, which is closely related to individuals' conflict monitoring. The emotional effects of motivation mainly occur in the later stage of response inhibition, which is closely related to the actual inhibition of the motor system.

### Limitations and Future Directions

Our findings mainly contribute to the growing body of literature on how violent virtual avatar may affect players. The results point to the role of chronic exposure to violent virtual avatar in explaining changes in response inhibition to emotional faces in players, which helps us to have a more comprehensive understanding of the impact of violent avatar use on players' cognition, deepen our understanding of the relationship between violent media and players' brain and the occurrence of aggressive behaviors. There are also some defects in this study. First, this study only explored the effects of long-term exposure to virtual avatars on players' response inhibition in violent video games. Since no causality can be inferred from our correlational findings, further study requires an experimental study to investigate any causal link between violent avatar experience and inhibitory control to angry expressions, such as the impact of short-term exposure to violent avatars on players' response inhibition to angry expressions. Second, virtual avatars include visual image (such as justice or evil) and meaning image (such as prosocial or antisocial; Heng et al., 2017; Heng et al., 2018), visual image refers to the visual form presented by the avatar appearance (such as gender, clothing), expression (such as smile), and nonverbal communication (such as gestures; McCreery et al., 2011). Meaning image refers to the image that describes the virtual avatar as prosocial or antisocial through the background narrative (Heng et al., 2017). Do different types of virtual avatars have the same effect on players' response inhibition? Further study requires further discussion on this issue. Third, this study only selected freshmen as the research objects, and further research needs to select middle school and high school adolescents as the research objects to increase the generalization of the results of this study. Finally, the Violent Virtual Avatar Experience measure has some limitations. Participants were asked to self-evaluate avatar violence. Due to a potential desensitization of participants highly exposed to violent video games, they could evaluate such avatars as less violent compared to less exposed participants. Further research could employ more objective tools for assessing violent content, such as the Pan European Game Information (PEGI) ratings.

### Conclusion

These results suggest that facing angry expression players with high violent virtual avatar experience show superiority in response inhibition, and this superiority exists in the later stage of response inhibition, which is closely related to the actual inhibition of the motor system.

## Notes

**Publication Ethics:** Ethical approval was obtained from the Institutional Review Board of the Xinxiang Medical University.

**Authorship:** Lijun Sun: Writing – review & editing, Supervision, Funding acquisition, Data curation, Conceptualization. Xuelian Li: Formal analysis, Writing – original draft. Shiyi Li: Data curation, Investigation. Xue Zhang: Data curation, Investigation. Yajing Si: Data curation. Hongxing Zhang: Supervision, Funding acquisition.

**Open Science:** Open Data: Complete data for all studies are presented on the Open Science Framework (OSF) website: https://osf.io/nhzd5/ (Zhang, 2026).
